# Planetary, inertia–gravity and Kelvin waves on the *f*‐plane and β‐plane in the presence of a uniform zonal flow

**DOI:** 10.1002/qj.4107

**Published:** 2021-06-21

**Authors:** Yair De‐Leon, Itzhak Fouxon, Chaim I. Garfinkel, Nathan Paldor

**Affiliations:** ^1^ Fredy and Nadine Herrmann Institute of Earth Sciences The Hebrew University of Jerusalem Jerusalem Israel

**Keywords:** PV sources, trapped waves, inertia‐gravity waves, Rossby waves, Kelvin wave, Doppler shifting

## Abstract

A linear wave theory of the Rotating Shallow‐Water Equations (RSWE) is developed in a channel on the midlatitude *f*‐plane or β‐plane in the presence of a uniform mean zonal flow that is balanced geostrophically by a meridional gradient of the fluid surface height. Here we show that this surface height gradient is a potential vorticity (PV) source that generates Rossby waves even on the *f*‐plane similar to the generation of these waves by PV sources such as the β‐effect, shear of the mean flow and bottom topography. Numerical solutions of the RSWE show that the resulting Rossby, Poincaré and “Kelvin‐like” waves differ from their counterparts without mean flow in both their phase speeds and meridional structures. Doppler shifting of the “no mean‐flow” phase speeds does not account for the difference in phase speeds, and the meridional structure is often trapped near one of the channel's boundaries and does not oscillate across the channel. A comparison between the phase speeds of Rossby waves of the present theory and those of the Quasi‐Geostrophic Shallow‐Water (QG‐SW) theory shows that the former can be 2.5 times faster than those of the QG‐SW theory. The phase speed of “Kelvin‐like” waves is modified by the presence of a mean flow compared to the classical gravity wave speed, and furthermore their meridional velocity does not vanish. The gaps between the dispersion curves of adjacent Poincaré modes are not uniform but change with the zonal wave number, and the convexity of the dispersion curves also changes with the zonal wave number. These results have implications for the propagation of Rossby wave packets: QG theory overestimates the zonal group velocity.

## Graphical abstract

The article analyses the effect that a mean zonal flow has on all wave types of the rotating shallow‐water equations in a channel in addition to the Doppler‐shift of the waves' phase speeds. The primary additional effect of the mean flow originates from the mean tilt of the layer's surface that balances geostrophically the Coriolis force associated with the mean flow. On the *f*‐plane this source of stretching vorticity generates planetary (Rossby) waves and on the *β*‐plane it can counterbalance planetary waves that originate from the gradient of planetary vorticity. The figure shows the dispersion relation, *ω*(*k*), of Rossby waves on the *f*‐plane in a narrow channel (width *L*) in the presence of a mean flow where *U* is the mean dimensional velocity scaled on *fL* and *α* = (*R*d/*L*)^2^ where *R*d is the radius of deformation.

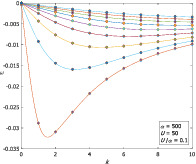

## 1 INTRODUCTION

Wave solutions of the linearized primitive equations form the basis of our understanding of atmospheric and oceanic dynamics, as they provide a simple description of how variability in one region can launch waves that affect other regions. The classical theory of waves in the ocean or atmosphere is derived from the linearized Rotating Shallow‐Water Equations (RSWE, hereafter) either on the *f*‐plane, where the Coriolis parameter is constant, or on the β‐plane where the Coriolis parameter varies linearly with latitude. This theory yields various types of wave, such as inertia–gravity (Poincaré), Kelvin and Rossby waves, where some of the wave types require particular configurations (e.g. walls for Kelvin waves and the β‐effect for Rossby waves).

In this classical theory on the *β*‐plane the Coriolis parameter is assumed constant even though its derivative, *β*, does not vanish. Thus, while the Rossby waves' frequency vanishes identically when *β* is set equal to 0, their amplitude in this theory does not vanish but varies harmonically (i.e. oscillatory) with latitude, as in equations that have no latitude‐dependent coefficients. However, numerical solutions of the associated eigenvalue equation in wide channels have shown that the waves' amplitudes are trapped near the equatorward channel wall and do not vary harmonically (Paldor *et al*., 2007; Gildor *et al*., 2016). In a series of recent articles, a new theoretical approach was developed whereby the Coriolis parameter is accounted for consistently in the linear RSWE on the *β*‐plane. For zonally propagating waves this new approach yields a second‐order (Schrödinger) differential equation. The analytic solutions obtained from this equation yield explicit expressions for the dispersion relations of Rossby waves as well as inertia–gravity waves and for their meridional amplitude structure, both of which differ appreciably from the classical solutions. On the midlatitude *β*‐plane, the new approach yields trapped waves instead of oscillatory (harmonic) waves and the phase speed and group velocity of long trapped Rossby waves exceed those of the classical harmonic theory by a factor that can be more than 2 in wide meridional domains (Paldor *et al*., 2007; Paldor and Sigalov, 2008; Gildor *et al*., 2016). These previous works considered the RSWE with no mean flow, and in this study we relax this restriction and consider how a mean flow changes the phase speed and meridional structure of linear waves.

From a potential vorticity (PV) perspective, planetary (Rossby) waves originate from potential vorticity gradients. Traditionally the PV gradient is associated with either variation of the Coriolis parameter with latitude (on the β‐plane) where the planetary vorticity varies with latitude, or variation in the height field by a sloping bottom (i.e. topographic Rossby waves) or by shear of a non‐uniform mean zonal current (Pedlosky, 1987). In the present study, we introduce Rossby waves on a flat‐bottom *f*‐plane that originate from a sloping *surface* that originates from the geostrophic relation between the mean height field and the zonal mean flow. This mean PV gradient, which appears to have been overlooked in previous studies, applies even when the mean flow is *uniform*, that is, in the absence of a mean relative vorticity. We construct an accurate linear theory based on the RSWE on the *f*‐plane or *β*‐plane and solve these equations numerically and, in some limits, also analytically by neglecting certain terms. We also show that a uniform mean flow affects the dispersion relation of the waves by more than a straightforward Doppler shift. In addition, the uniform mean flow affects the meridional structure of the eigenfunctions of all waves, including that of Rossby waves that prevail in this case on the *f*‐plane.

The vorticity view naturally leads to the quasi‐geostrophic equation of the RSWE, known as the Quasi‐Geostrophic Shallow‐Water theory (QG‐SW: Vallis, 2017). The resulting solutions of this theory also include more than just Doppler shift but because the divergence equation is filtered out in QG theories, a complete force balance cannot be achieved. Furthermore, the QG‐SW theory precludes inertia–gravity and Kelvin waves, and while it yields explicit expressions for the dispersion relation of Rossby waves, the meridional amplitude structure in this theory is harmonic. These results of the QG‐SW theory are examined in the present study.

The article is organized as follows: in Section 2 we introduce the governing equations with uniform mean flow. We focus on the *f*‐plane in Section 3 and present analytical and numerical results. In Section 4 we examine the effect of a uniform mean flow on waves on the *β*‐plane, and the article ends in Section 5 with a discussion of its findings and the implications for atmospheric Rossby wave packets.

## 2 GOVERNING EQUATIONS

### 2.1 Shallow‐water equations with mean zonal flow

The RSWE are employed here in a zonal channel of width 2*L* where *u* and *v* are the velocities in the zonal (*x*) and meridional (*y*) directions, respectively. The non‐dimensional form of the RSWE is obtained from the dimensional equations (see e.g. Vallis, 2017) as follows: *L* scales the horizontal coordinates *x* and *y*; $1/f_{0}=1/\left(2\text{Ωsin}\phi_{0}\right)$ is the time‐scale (where Ω is Earth's rotation frequency and $\phi_{0}$ is the mean latitude); $f_{0}L=2\Omega L\sin\phi_{0}$ is the velocity scale and *H* (the mean height of the fluid layer) is the scale for *η*, the deviation of total height from *H*. The Coriolis frequency is assumed to vary linearly with *y*, that is, in dimensional form, $f=f_{0}+\beta y=2\text{Ωsin}\phi_{0}+\frac{2\text{Ωcos}\phi_{0}}{a}y$ (where *a* is Earth's radius) and is scaled on $f_{0}$ so its non‐dimensional form is $f=1+\beta y=1+\cot\phi_{0}\frac{L}{a}y$ (so in the non‐dimensional system $\beta =\cot\phi_{0}\frac{L}{a}$). Aside from β, the single non‐dimensional parameter that incorporates all dimensional parameters (including the gravitational acceleration, *g*) of the dimensional equations is $\alpha =\frac{\mathrm{gH}}{{\left(2\Omega L\sin\phi_{0}\right)}^{2}},$ defined as the square of the ratio between the radius of deformation, $R_{d}=\frac{\sqrt{\mathrm{gH}}}{f_{0}}=\frac{\sqrt{\mathrm{gH}}}{2\text{Ωsin}\phi_{0}}$, and half the channel width, *L* (note: since $f_{0}L$ is the velocity scale, *Fr* $=\left(f_{0}L\right)/\sqrt{\mathrm{gH}}$ is the Froude number based on the velocity scale $f_{0}L$ so $\alpha ={\mathrm{Fr}}^{-2}$). The non‐dimensional boundary conditions of no normal flow at the channel walls are v(y=±1)=0.

We assume that a mean zonal flow exists accompanied by a height profile so *u* and η can be written as the sum of a mean and perturbed variables:

$$u\left(x,y,t\right)=\bar{u}+u^{\prime }\left(x,y,t\right)\;\text{and}\;\eta \left(x,y,t\right)=\bar{\eta }\left(y\right)+\eta^{\prime }\left(x,y,t\right).$$

This formulation applies to any $\bar{u}=\bar{u}\left(y\right)$ but here we assume that $\bar{u}$ is constant. Since there is no mean meridional velocity, $v=v^{\prime }$. We also denote $1+\bar{\eta }=\bar{h}$ where $\bar{h}$ is the mean (meridional) height distribution in the atmosphere/ocean.

The leading order (mean) terms in the non‐dimensional RSWE yield the geostrophic relation:

(1) $$f\bar{u}=-\alpha \frac{d\;\bar{\eta }}{dy}=-\alpha \frac{d\bar{h}}{dy},\;i.e.\;\frac{d\bar{h}}{\mathrm{dy}}=-\frac{f}{\alpha }\bar{u}.$$

For the constant $\bar{u}=U$ assumed here, an integration of Equation (1) with respect to *y* yields:

(2) $$\begin{array}{cc} \bar{h} & =-\int \frac{1}{\alpha }f\bar{u}\mathrm{dy}=-U/\alpha \int \left(1+\beta y\right)\mathrm{dy}\\ & =-U/\alpha \left(y+\frac{1}{2}\beta y^{2}\right)+A, \end{array}$$

where *A* is an integration constant to be determined next. The velocity scale of $f_{0}L$ implies that the non‐dimensional *U* is also the Rossby number, $R_{0}$, of the mean flow so *U*/*α* in Equation (2) equals $R_{0}{\mathrm{Fr}}^{2}$.

In the constant density theory developed here, conservation of mass is tantamount to the conservation of volume. Thus, the volume per unit (zonal) length that is, the cross‐channel area, $\;\int_{-1}^{1}\bar{h}\mathrm{dy}$, should be identical for any $\bar{u}$. In the absence of mean flow, that is, when $\bar{u}=0$, $\frac{d\bar{h}}{\mathrm{dy}}=0$ so $\bar{h}=1$ which implies that the cross‐channel area $\int_{-1}^{1}\bar{h}\mathrm{dy}=2.$ The conservation of volume (mass) per unit length when $\bar{u}\neq 0$, that is, when $\frac{d\bar{h}}{\mathrm{dy}}\neq 0$, allows us to determine the constant *A* uniquely by substituting $\bar{h}$ of Equation (2) into $\int_{-1}^{1}\bar{h}\mathrm{dy}=2$, which implies: $A=1+\frac{\beta }{6}U/\alpha$. Substituting this expression for *A* in Equation (2) yields:

(3) $$\bar{h}=1+U/\alpha \left(\frac{\beta }{6}-y-\frac{1}{2}\beta y^{2}\right).$$

Now, since on the midlatitude *β*‐plane *β* < 1 (note: $\beta =\cot\phi_{0}\frac{L}{a}$), $\bar{h}\left(y\right)$ decreases monotonically for *U* > 0 and increases monotonically for *U* < 0, so the minimal value of $\bar{h}=1+\bar{\eta }$ occurs at y=1 for *U* > 0 and at y=−1 for *U* < 0 (and vice versa in the Southern Hemisphere where *β* < 0). Requiring $\bar{h}$ to be positive everywhere implies:

(4) $$\begin{array}{cc} U & <\frac{\alpha }{1+\frac{\beta }{3}}\;\text{when}\;U>0;\\ -U & <\frac{\alpha }{1-\frac{\beta }{3}}\;\text{when}\;U<0. \end{array}$$

This condition determines the maximal value of U for given values of αandβ and it implies the existence of an upper bound of the speed, which is lower for an easterly mean wind than for a westerly mean wind (and the opposite in the Southern Hemisphere). On the *f*‐plane, where *β* = 0, the two upper bounds of the speed are identical. Note that no such bound exists in the QG‐SW theory since this theory does not address force balances.

We now linearize the RSWE about the geostrophic mean variables, $U,\bar{h}$ retaining only linear terms in $u^{\prime },v^{\prime },\eta^{\prime },$ which yields:

(5) $$\left\{\begin{array}{l} \frac{\partial u^{\prime }}{\partial t}+U\frac{\partial u^{\prime }}{\partial x}-{\mathrm{fv}}^{\prime }=-\alpha \frac{\partial \eta^{\prime }}{\partial x}\\ \frac{\partial v^{\prime }}{\partial t}+U\frac{\partial v^{\prime }}{\partial x}+fu^{\prime }=-\alpha \frac{\partial \eta^{\prime }}{\partial y}\\ \frac{\partial \eta^{\prime }}{\partial t}+\bar{h}\left(\frac{\partial u^{\prime }}{\partial x}+\frac{\partial v^{\prime }}{\partial y}\right)+U\frac{\partial \eta^{\prime }}{\partial x}+v^{\prime }\frac{\partial \bar{h}}{\partial y}=0. \end{array}\right.$$

In the traditional wave theory of the RSWE (as well as in the QG‐SW theory) the variation in *y* is assumed to have the harmonic form $e^{i\mathrm{ly}}$. However, since *f* and $\bar{h}$ in system (5) are both *y*‐dependent we leave the determination of the *y*‐dependence of the solution to a later stage. Accordingly, we assume a zonally propagating wave with unspecified *y*‐dependent amplitude: $\left[\begin{array}{c} u^{\prime }\\ \begin{array}{c} v^{\prime }\\ \eta^{\prime } \end{array} \end{array}\right]=\left[\begin{array}{c} u\left(y\right)\\ \begin{array}{c} v\left(y\right)\\ \eta \left(y\right) \end{array} \end{array}\right]e^{i\left(\mathrm{kx}-\omega t\right)}$ where *k* is the (non‐dimensional) zonal wave number and ω is the (non‐dimensional) frequency (note that hereafter the unprimed variables denote the *y*‐dependent amplitudes of the perturbation variables). Substituting this wave form in system (5), rearranging and defining: $V=\frac{iv}{k}$, yields the matrix form:

(6) $$\left[\begin{array}{ccc} 0 & \left(1+\beta y\right) & \alpha \\ \frac{\left(1+\beta y\right)}{k^{2}} & 0 & \frac{\alpha }{k^{2}}\frac{d}{\mathrm{dy}}\\ \bar{h} & -\left(\frac{d\bar{h}}{\mathrm{dy}}+\bar{h}\frac{d}{\mathrm{dy}}\right) & 0 \end{array}\right]\left[\begin{array}{c} u\\ V\\ \eta \end{array}\right]\;=\;\left(C\;-\;U\right)\left[\begin{array}{c} u\\ V\\ \eta \end{array}\right]\;=\;c\left[\begin{array}{c} u\\ V\\ \eta \end{array}\right],$$

where C=ω/k is the (non‐dimensional) phase speed and c=(C−U) is the Doppler‐shifted phase speed. For *y*‐dependent $\bar{u}$ the term $-{\bar{u}}_{y}$ should be added to the second element of the first row in this matrix.

The three‐dimensional set (6) can be solved numerically as a differential eigenvalue problem (in which *c* is the eigenvalue) using the Chebyshev collocation method (see more details in De‐Leon and Paldor (2009)). Alternatively, since the first (i.e. *u*) equation has no *y*‐derivatives, *u* can be eliminated from this set by expressing it as the following linear combination of *V* and η:

(7) $$u=\frac{\left(1+\beta y\right)V+\alpha \eta }{c}.$$

Substituting this relation in the other two equations of system (6) yields the two‐dimensional differential set for Vandη:

(8) $$\frac{d}{\mathrm{dy}}\left[\begin{array}{c} V\\ \eta \end{array}\right]=\frac{1}{c}\left[\begin{array}{cc} \left(1+\beta y\right)-\frac{c}{\bar{h}}\frac{d\bar{h}}{\mathrm{dy}} & \alpha -\frac{1}{\bar{h}}c^{2}\\ \frac{1}{\alpha }\left(k^{2}c^{2}-{\left(1+\beta y\right)}^{2}\right) & -\left(1+\beta y\right) \end{array}\right]\left[\begin{array}{c} V\\ \eta \end{array}\right].$$

This set, too, can be solved using a shooting method (see details in Paldor and Dvorkin, 2006 and De‐Leon and Paldor, 2009). A match between the values of *c* and the form of the resulting functions (u(y),V(y),η(y)) that solve the two (different) differential systems Equations (6) and (8) verifies the accuracy of our numerical results.

## 3 MEAN FLOW ON THE *f*‐PLANE: THE EMERGENCE OF ROSSBY WAVES AND THE MODIFIED KELVIN AND POINCARÉ WAVES

Our analysis of the wave characteristics in the presence of mean flow begins by focusing on the *f*‐plane obtained by setting β=0 in Equations (3), (4), (5), (6), (7), (8). In this greatly simplified case, explicit approximate expressions can be derived for solutions of the eigenvalue problem.

### 3.1 Analytical approximations

On the *f*‐plane the mean state relation, Equation (1), becomes: $\frac{d\bar{h}}{\mathrm{dy}}=-U/\alpha$, the perturbation set (8), with $\bar{h}=1-(U/\alpha )y$ (Equation (3)), becomes:

(9) $$\frac{d}{\mathrm{dy}}\left[\begin{array}{c} \begin{array}{c} V \end{array}\\ \begin{array}{c} \eta \end{array} \end{array}\right]=\frac{1}{c}\left[\begin{array}{cc} 1+\frac{c}{1-(U/\alpha )y}U/\alpha & \;\alpha -\frac{c^{2}}{1-(U/\alpha )y}\\ \frac{k^{2}c^{2}-1}{\alpha } & \;-1 \end{array}\right]\left[\begin{array}{c} \begin{array}{c} V \end{array}\\ \begin{array}{c} \eta \end{array} \end{array}\right],$$

and condition (4) becomes: −1<U/α<1. The reader is reminded that with our scaling, $U/\alpha =R_{0}{\mathrm{Fr}}^{2}$, where $R_{0}$ is the Rossby number of the mean flow and *Fr* is the Froude number based on the velocity scale $f_{0}L$.

The degenerate solution, known as Kelvin waves, obtained by setting *V*(*y*) = 0 identically in the RSWE with no mean flow, does not exist here since V(y)≡0 implies $\frac{\mathrm{dV}}{\mathrm{dy}}\equiv 0$ and when these are substituted in the first equation of (9), it becomes: $\left(\alpha -\frac{c^{2}}{1-y\;U/\alpha }\right)\eta =0$. For η≠0 this solution implies $c^{2}=\alpha -\mathrm{Uy}$ (which degenerates to $\;c=\pm \sqrt{\alpha }$, the Kelvin wave's phase speed, for U=0), that is, c=c(y), in which case additional terms proportional to dc/dy should be included in sets (6) and (9); however, such additional terms involving c(y) are inconsistent with the derivation of the differential set by separating the variables. The conclusion is that the V(y)≡0 assumption does not yield solutions of system (9) when U≠0. Therefore, in the presence of a mean flow the existence and characteristics of Kelvin waves (discussed in the next section) differ qualitatively from those derived for *U* = 0.

To advance, we transform the second‐order system (9) to a single second‐order equation. Since the boundary conditions are expressed in terms of *V* [i.e. V(y=±1)=0], solutions can be more easily obtained by deriving a second‐order equation for *V* by eliminating η. We thus differentiate the first equation (for dV/dy) of (9) with respect to *y* and substitute in the resulting equation the expressions for dη/dy from the second equation of (9) and that for η from the first equation of (9). This yields the single, second‐order, equation for V:

(10) $$\begin{array}{cc} & \left(1-yU/\alpha \right)\frac{d^{2}V}{dy^{2}}-\left(1-\frac{c^{2}}{\alpha \left(1-yU/\alpha \right)-c^{2}}\right)U/\alpha \frac{\mathrm{dV}}{\mathrm{dy}}\\ & +\left(E+k^{2}yU/\alpha -\frac{1+\frac{c}{U}}{\left(\left(1-yU/\alpha \right)-\frac{c^{2}}{\alpha }\right)}{\left(U/\alpha \right)}^{2}\right)V=0, \end{array}$$

where $E=-\left(k^{2}-\frac{k^{2}c^{2}}{\alpha }+\frac{1}{\alpha }+\frac{1}{c}U/\alpha \right)$. All *y*‐dependent terms in Equation (10) are proportional to *U*/*α* and since −1<U/α<1 (see Equation (4)) and |y|≤1 the equation has no singular points unless $\alpha -U\leq c^{2}\leq \alpha +U$, and this special case will be treated in the next subsection. These considerations suggest that a simple and regular solution can exist at sufficiently small *U*/α which is the starting point of our analysis.

#### 3.1.1 Analytic solutions for small *U*/*α*

The assumption *U*/*α* ≪1 implies the following ramifications that apply to Equation (10):

1. The coefficient of $\frac{d^{2}V}{dy^{2}}$ (i.e. 1−(U/α)y) becomes 1,
2. The $k^{2}(U/\alpha )y$ term in the coefficient of *V* can be neglected for not‐too‐large *k*,
3. $U/\alpha \frac{\mathrm{dV}}{\mathrm{dy}}$ can be neglected, that is, $\frac{\mathrm{dV}}{\mathrm{dy}}$ is not singular (this is confirmed mainly by the numerical results),
4. The $\frac{1+\frac{c}{U}}{\left(\left(1-(U/\alpha )y\right)-\frac{c^{2}}{\alpha }\right)}{\left(U/\alpha \right)}^{2}$ term in the coefficient of *V* can be neglected.

Under these assumptions Equation (10) becomes a constant‐coefficient equation of the form:

(11) $$\frac{d^{2}V}{dy^{2}}+\mathrm{EV}=0.$$

In contrast to Equation (10), Equation (11) is a Schrödinger equation that defines a complete discrete set of eigenfunctions, $V_{n}\left(y\right)$, (where *n* is the mode number, determined by the number of internal zero‐crossings of the eigenfunction) and eigenvalues (“energies”), $E_{n}$, that are related to the phase speeds, $c_{n}$, by:

(12) $$k^{2}c_{n}^{3}-\left(1+\alpha k^{2}+\alpha E_{n}\right)c_{n}-U=0,$$

i.e. the phase speeds are the roots of this cubic equation.

The detailed derivation of Equation (11) described above can also be derived heuristically in an analogous fashion to the traditional derivation of wave theory on the β‐plane where one assumes that $f=f_{0}=\text{constant}$ but $\frac{\mathrm{df}}{\mathrm{dy}}=\beta \neq 0$ and gets a constant coefficients second‐order equation (see Paldor *et al*., 2007). In the current set‐up one can assume $\bar{h\;}$= 1 but $\frac{d\bar{h}}{\mathrm{dy}}=-U/\alpha \neq 0$ for small *U*/*α* in (9) and derive the analytically solvable Equation (11).

The solutions of Equation (11) are $V_{n}\left(y\right)=V_{0}\sin\left(\sqrt{E_{n}}y+\theta \right),$ where $V_{0}$ is an arbitrary amplitude and θ is an undetermined phase. Given the boundary conditions V(y=±1)=0 the energies are given by $E_{n}=\frac{\pi^{2}{\left(n+1\right)}^{2}}{4}$ where *n* = 0, 1, 2, 3,.., (note that $E_{n}=0$ yields *V* = 0 everywhere) and the three roots of Equation (12) yield three phase speeds for each *n*. To ensure that the eigenfunction satisfies the boundary conditions, its phase should be $\theta_{n}=\frac{\left(n+1\right)\pi }{2}$ so the eigenfunctions are: $V_{n}\left(y\right)=V_{0}\sin\left(\frac{\left(n+1\right)\pi }{2}\left(y+1\right)\right)$. Substituting this solution for *V*_*n*_(*y*) in the first equation of (9) yields $\eta_{n}\left(y\right)=A\sin\left(\frac{\left(n+1\right)\pi }{2}\left(y+1\right)\right)+B\cos\left(\frac{\left(n+1\right)\pi }{2}\left(y+1\right)\right)$ where *A* and *B* are amplitudes that depend on $c_{n}$. Thus, for a given *n*, $\eta_{n}\left(y\right)$ is not identical for all three values of $c_{n}$ while *V*_*n*_(*y*) is! (see a similar scenario in De‐Leon and Paldor, 2009).

Approximate expressions for the phase speeds of the slowly propagating Rossby waves are obtained by neglecting the cubic term, $k^{2}c_{n}^{3}$, relative to the linear term (in $c_{n}$) in Equation (12), so for $E_{n}=\frac{\pi^{2}{\left(n+1\right)}^{2}}{4}$ the phase speed of Rossby waves is:

(13) $$c_{n}^{\text{Rossby}}\approx \frac{-U/\alpha }{k^{2}+E_{n}+1/\alpha }=\frac{-U/\alpha }{k^{2}+\pi^{2}{\left(n+1\right)}^{2}/4+1/\alpha }.$$

This expression for the Doppler‐shifted phase speed (see the discussion following Equation (6)) of Rossby waves is similar to the corresponding expression in the classical β‐plane theory, but with one key difference: here U/α plays the role of β (both of which can be negative in the non‐dimensional equations). In other words, Rossby waves can exist in a constant zonal flow on the *f*‐plane due to the sloping surface of the height field.

Approximate expressions for the phase speeds of the fast‐propagating Poincaré waves are obtained when the free term (*U*) is neglected in Equation (12) and the resulting equation is divided through by $c_{n}$, which yields:

(14) $$\begin{array}{cc} {\left(c_{n}^{\text{Poincar}\overset{'}{e}}\right)}^{2} & \approx \left(\frac{1}{k^{2}}+\alpha \left(1+\frac{E_{n}}{k^{2}}\right)\right)\\ & =\left(\frac{1}{k^{2}}+\alpha \left(1+\frac{\pi^{2}{\left(n+1\right)}^{2}/4}{k^{2}}\right)\right). \end{array}$$

The r.h.s. of this expression for the Doppler‐shifted phase speed is identical to that of Poincaré waves on the *f*‐plane without mean flow. In the next section we examine the accuracy of the approximate expressions, Equations 13 and 14, by comparing them with the roots of the cubic Equation (12) and with exact numerical solution of (9) or (6).

For large *k* (but small *U*/α), the term $k^{2}(U/\alpha )y$ cannot be neglected in the coefficient of *V* in Equation (10) while the other neglected terms proportional to (U/α)y≪1 can. Transformation of the independent variable, *y*, to $z\left(y\right)=-{\left(k^{2}U/\alpha \right)}^{-\frac{2}{3}}\left(E+k^{2}(U/\alpha )y\right)$ transforms Equation (10) to an Airy equation, $\frac{d^{2}V}{dz^{2}}-\mathrm{zV}=0,$ whose general solution is a linear combination of two functions Ai(z) and Bi(z). Both Ai(z) and Bi(z) oscillate for z < 0, but while Ai(z) decays to zero for z > 0, Bi(z) grows there faster than exponentially (see Paldor and Sigalov, 2008 for more details). To satisfy the boundary conditions (V(y=±1)=0) we set y=−1 at z=2 where Ai(z=2) is sufficiently close to zero so the contribution of Bi(z) must be negligible. The boundary condition at y=1 is satisfied by setting z(y=1) to be one of the zeros of Ai(z) denoted by $\left(-\zeta_{n}\right)$ (as in Paldor and Sigalov, 2008). This determines the energy levels as:

(15) $$E_{n}={\left(k^{2}U/\alpha \right)}^{\frac{2}{3}}\zeta_{n}-k^{2}U/\alpha ,$$

and the corresponding eigenfunctions as:

(16) $$V_{n}\left(z\left(y\right)\right)=\mathrm{Ai}\left(z\left(y\right)\right)=\mathrm{Ai}\left({\left(k^{2}U/\alpha \right)}^{\frac{1}{3}}\left(1-y\right)-\zeta_{n}\right).$$

The neglect of the contribution of Bi(z) to the solution and satisfying $V_{n}\left(y=-1\right)\to 0$ are both justified provided z(y=−1)≥2, that is, for $k^{2}U/\alpha >{\left(\frac{2+\zeta_{n}}{2}\right)}^{3}$, so for given *U*/*α* as *n* increases (and with it $\zeta_{n}$), the value of *k* should also increase to ensure the validity of the approximation by the Airy function. Approximate expressions for the phase speeds of Rossby and Poincaré waves are obtained by substituting the expression for $E_{n}$ of Equation (15) in the middle expressions in Equations (13) and (14). The transformation of the eigenfunction structure from harmonic to Airy‐like when *k* increases is verified in the next subsection. To the best of our knowledge, this transformation is missed by previous wave theories.

Additional “Kelvin‐like” solutions of (9), that are *not* derived from solutions of the eigenvalue problem, Equation (11), might be obtained for values of *c* in the range $\alpha -U\leq c^{2}\leq \alpha +U$ for which the denominator of two of the terms in Equation (10) has a singular point at some −1<y<1 and not necessarily at y=0, and for which the coefficient of η in the first equation of (9) vanishes. The exact eigenvalues and the corresponding eigenfunctions are determined numerically in the next subsection. These additional wave solutions are termed “Kelvin‐like” and “anti‐Kelvin‐like” waves since $c^{2}\approx \alpha$, as in Kelvin waves, but unlike Kelvin waves, V(y)≢0. These solutions solve the exact systems (6) and (9) but are not approximated by the Schrödinger equation, Equation (11), so they are not part of the complete set of solutions and should not be expected to satisfy the properties of such a set.

Clearly, as *U* increases, a larger range of c(*k*) values can satisfy the inequality $\alpha -U\leq c^{2}\left(k\right)\leq \alpha +U$ so in addition to generating the “Kelvin‐like” mode this singularity affects the Poincaré and Rossby modes provided the eigenfunction does not vanish at the singular point.

### 3.2 Numerical solutions

**FIGURE 1:**
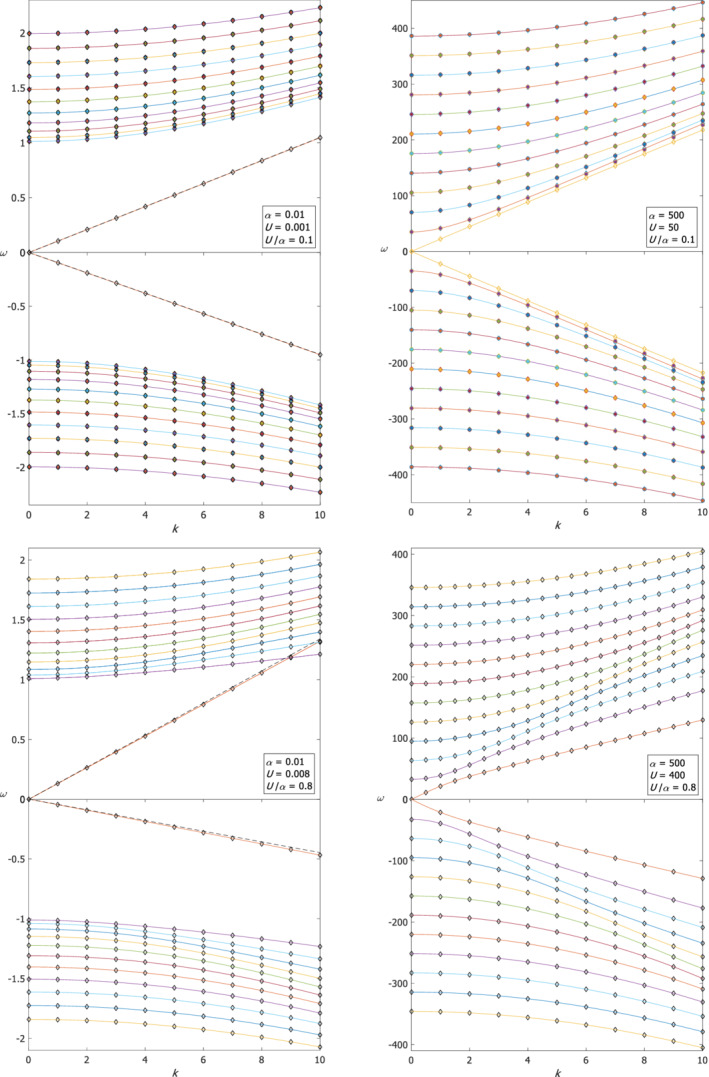
The dispersion relations of the Poincaré waves and two additional “Kelvin‐like” and “anti‐Kelvin‐like” waves in a wide/narrow channel (left/right panels) for small/large U/α (upper/lower panels). Diamonds represent the ω‐values obtained by numerical solution of set (9) while the curves are the spline‐interpolation of these values. Dashed lines are analytic approximation of the “Kelvin‐like” and “anti‐Kelvin‐like” dispersion relations. Circles are the ω‐values of positive/negative Poincaré waves obtained by the largest/smallest root of Equation (12) for each *k* and *n*, and asterisks are the ω‐values obtained by the approximate expression, Equation (14), for each *k* and *n* [Colour figure can be viewed at wileyonlinelibrary.com]

**FIGURE 2:**
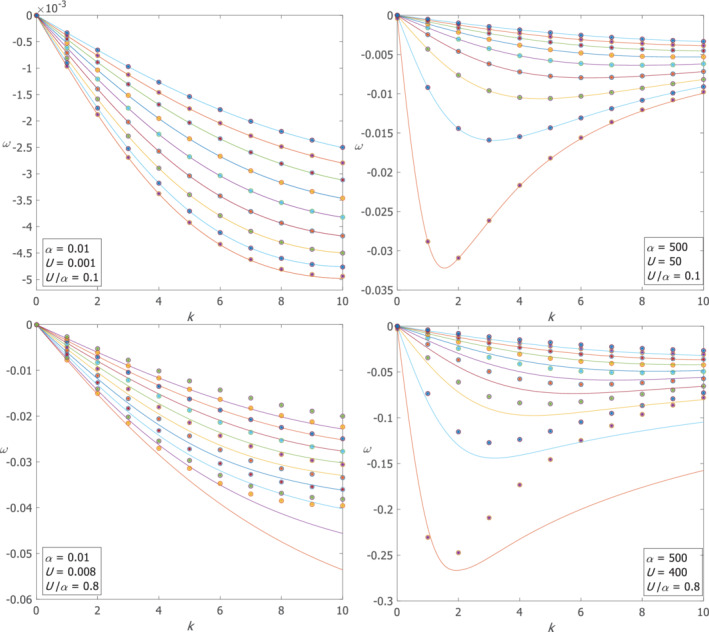
The dispersion relations of the Rossby waves in in a wide/narrow channel (left/right panels) for small/large U/α (upper/lower panels). Lines are spline‐interpolations of the ω‐values obtained by numerical solution of set (9), circles are the ω‐values obtained by the smallest (in absolute value) root of Equation (12) for each *k* and *n*, and asterisks are the ω‐values obtained by the approximation of Equation (13) for each *k* and *n* [Colour figure can be viewed at wileyonlinelibrary.com]

The dispersion relations of the first 11 meridional modes (i.e. *n* = 0 to 10) of both positive and negative Poincaré waves are shown in Figure 1 and the dispersion relations of the first 9 meridional modes (i.e. *n* = 0 to 8) of Rossby waves are shown in Figure 2. In Figures 1 and 2, the two upper panels are for small *U*/α and the two lower panels are for large *U*/α. The two left panels in Figures 1 and 2 are for small α=0.01 (i.e. a wide channel, since the channel width, 2*L*, is larger than *R*_d_) and the two right panels are for large α=500 (i.e. a narrow channel). Diamonds represent the exact ω‐values obtained by numerical solutions of system (9) and the connecting continuous curves are the spline‐interpolation of these values. Circles are the ω‐values of positive/negative Poincaré waves (Rossby waves) obtained by the largest/smallest (smallest in absolute value) root of Equation (12) for each *k* and *n*, and asterisks are the ω‐values obtained by the approximate expression, Equation (14) (Equation (13)) for each *k* and *n*.

**FIGURE 3:**
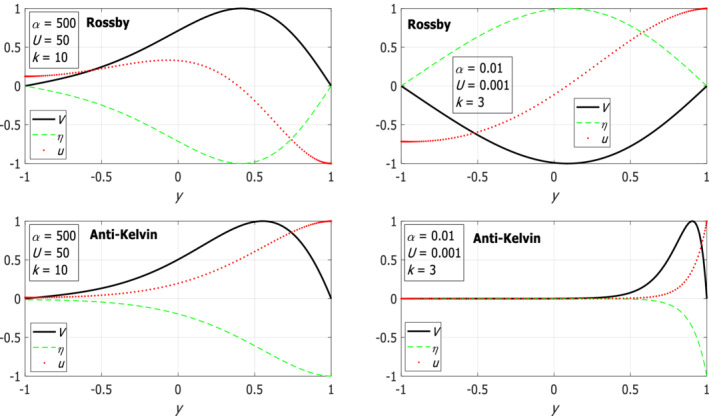
Examples of the *V*‐eigenfunctions of Rossby (upper panels) and “anti‐Kelvin‐like” (lower panels) waves on the *f*‐plane for small *U*/α and for the indicated two values of *k* (10 in left panels and 3 in right panels). Also shown are the corresponding variations of *u* and *η*. All eigenfunctions are normalized such that their maximum/minimum amplitude is 1/−1 [Colour figure can be viewed at wileyonlinelibrary.com]

Examples of the eigenfunctions (normalized such that the maximum/minimum amplitude is 1/−1) of Rossby (*n* = 0) and the “anti‐Kelvin‐like” waves are shown in Figure 3 for small *U*/α for both narrow (large α; left panels) and wide (small α; right panels) channels. A detailed discussion of the results is provided below.

#### 3.2.1 Results: Small U/α=0.1

##### *A wide channel (small* α=0.01 *and* U=0.001)

The upper‐left panel of Figure 1 shows the dispersion relations of Poincaré waves where there is an excellent agreement of better than 1% between the three (one numerical and two analytic) estimates. For large values of *k* and small *n*, the ω‐values obtained by the Airy approximation are even closer to the exact numerical solution of (9). Also shown is the dispersion relation of the additional, “Kelvin‐like” solution, which is a solution of set (9) but not of the approximate eigenvalue problem Equation (11), so it is not approximated by any of the roots of Equation (12). Instead, the numerical dispersion curve of the “Kelvin‐like” mode is compared with the analytic value of ω=ck where $c=\sqrt{\alpha \left(1-yU/\alpha \right)}$ is evaluated at y=−1 (dashed line; see above in the paragraph following Equation (9)) since the η‐eigenfunction of this mode (not shown) decays exponentially within a short distance from the southern wall of the channel (i.e. near y=−1). Similarly, the numerical solution of the “anti‐Kelvin‐like” mode is compared with the value of $c=-\sqrt{\alpha \left(1-yU/\alpha \right)}$ evaluated at y=1 (dashed line) since the η‐eigenfunction of this mode decays with distance from the northern wall of the channel (i.e. y=1, see the eigenfunctions in the lower‐right panel of Figure 3). Thus, the dispersion relations of the “Kelvin‐like” and “anti‐Kelvin‐like” modes are not symmetric (however, for these values of α and U the difference between the two modes is small).

The dispersion relations of the Rossby waves are shown in the upper‐left panel of Figure 2 and here, too, there is a very good agreement between the exact numerical solutions and the analytical approximations.

Though the eigenvalues of Equation (9) are approximated very well by the eigenvalues of Equation (11) for all *k*, the associated eigenfunctions of Equation (9) are (nearly) harmonic only for small *k*. In contrast, at large *k* the symmetry in *y* is broken since the term $k^{2}yU/\alpha$ of Equation (10) becomes significant so the eigenfunctions are approximated by the Airy function, Equation (16); see the sample of eigenfunctions in the upper panels of Figure 3. Surprisingly, the *V*‐eigenfunctions of the Rossby and two Poincaré waves calculated from Equation (9) (that are classified based on the values of the corresponding phase speeds) are nearly identical for the same values of *k*. The obvious reason is that these three *V*‐eigenfunctions (two for Poincaré waves and one for Rossby waves) correspond to the same *V*‐eigenfunction of Equation (11) that yields the same energy value, *E*, for the three waves. The identity of *V*‐eigenfunctions of the three waves holds regardless of whether the value of *E* is calculated based on the harmonic approximation (where the $k^{2}yU/\alpha$ term is neglected) or from the Airy‐function approximation, Equation (16) (where the $k^{2}yU/\alpha$ term is retained). The results derived in this case are summarized in the upper‐left cell of Table 1.

**TABLE 1:** The main findings on the *f*‐plane for the four combinations of U,α

	Wide channel (small α=0.01)	Narrow channel (large α=500)
Small U/α	*V* of Rossby and Poincaré – Harmonic or trapped depending on *k* Kelvin‐like/anti‐Kelvin‐like waves decay near a channel wall with $c=\pm \sqrt{\alpha \left(1-yU/\alpha \right)}$ at y=∓1 (not symmetric)	*V* of Rossby, Poincaré *and Kelvin‐like* – Harmonic or trapped depending on *k* Nearly symmetric Kelvin/anti‐Kelvin waves with $c=\pm \sqrt{\alpha \left(1-yU/\alpha \right)}$ at some −1 ≤ *y* ≤ 1
Large U/α	*V* of Rossby and Poincaré – Trapped (maximum near y=1) Kelvin‐like/anti‐Kelvin‐like waves decay near a channel wall with $c=\pm \sqrt{\alpha \left(1-yU/\alpha \right)}$ at y=∓1 (not symmetric) Kelvin's dispersion curve crosses Poincaré modes Small gap between curves of adjacent Poincaré modes for small *k* values that fan out for large *k*	*V* of Rossby and Poincaré – Trapped (maximum near y=1) Symmetric Kelvin‐like/anti‐Kelvin‐like waves maximum amplitude near y=1 (but not decaying) with $c=\pm \sqrt{\alpha \left(1-yU/\alpha \right)}$ at some −1 ≤ *y* ≤ 1 Not clear whether Kelvin's dispersion curve crosses Poincaré modes or not Convexity of Poincaré modes changes with *k*

##### *A narrow channel (large* α=500andU=50)

For larger values of α (but same *U*/*α* =0.1 as in the wide channel case), the harmonic approximation yields highly accurate estimates but the estimates for the eigenfunctions are only accurate at small *k* values. Two additional modes exist that do not match the harmonic approximations, but in contrast to classical Kelvin and anti‐Kelvin modes they do not decay near either of the channel walls. These two additional modes have phase speed values of $c=\pm \sqrt{\alpha \left(1-yU/\alpha \right)}$ evaluated at some *y* between −1 and 1 that varies with *k* (in contrast to the constant phase speed of classical Kelvin waves that does not depend on *k*). The upper‐right panel of Figure 1 shows the dispersion relations of both the positive and negative Poincaré waves and the two additional modes which are nearly symmetric. The frequency gap between the curves of adjacent (small *n*) Poincaré modes slowly decreases with *k* as in the dispersion relation of Poincaré waves with no mean flow. The dispersion relations of Rossby waves in the upper‐right panel of Figure 2 are similar to those of the classical wave theory (i.e. without mean flow) on the β‐plane and they agree very well with the harmonic approximation. The eigenfunctions of both negative and positive Poincaré waves and of Rossby waves have their maximum at y=0 for small *k* (i.e. harmonic solution) and it moves toward y=1 as *k* increases (see the upper‐left panel of Figure 3). This is also the behaviour of the additional two modes, in contrast to classical Kelvin and anti‐Kelvin waves in which the η‐eigenfunctions decay on the opposite channel walls (see this behaviour in lower‐left panel of Figure 3). The results derived for this case are summarized in the upper‐right cell of Table 1.

In both narrow and wide channels, the *V* function (solid black line) of the “anti‐Kelvin‐like” wave does not vanish (lower panels of Figure 3), in contrast to that of the classical Kelvin waves (i.e. when *U* = 0).

#### 3.2.2 Results: Large *U*/α =0.8

For large *U*/*α*, Equation (10) cannot be approximated by Equation (11) and its harmonic solutions, so only numerical solutions can be obtained. Nevertheless, some of the properties of a Sturm–Liouville equation still exist, such as the discrete modes with increasing number of internal zero‐crossings of the eigenfunctions. However, the dispersion curves of the additional “Kelvin‐like” and “anti‐Kelvin‐like” modes cross the dispersion relation curves of Poincaré modes, that is, two modes with the same number of internal zero‐crossings can co‐exist when one of them is not part of the complete set of solutions. The eigenfunctions of Poincaré and Rossby waves are not harmonic but similar to the Airy function where their maximum shifts toward y=1 as *k* increases.

##### *A wide channel (small* α=0.01andU=0.008)

The lower‐left panel of Figure 1 shows the dispersion relations of both the positive and negative Poincaré waves and the two additional modes. The numerical dispersion curves of the “Kelvin‐like”and “anti‐Kelvin‐like” modes are compared with the analytic values of ω=ck where $c=\sqrt{\alpha \left(1-yU/\alpha \right)}$ with y=−1 for the “Kelvin‐like” mode and $c=-\sqrt{\alpha \left(1-yU/\alpha \right)}$ with y=1 for the “anti‐Kelvin‐like” mode (dashed lines). It is clear that the “Kelvin‐like” and “anti‐Kelvin‐like” modes do not have the same phase speed and that the “Kelvin‐like” wave crosses the first modes of the positive Poincaré waves. The gap between curves of adjacent small *n* modes of Poincaré waves is small for small *k* values and it fans out for large *k*, in contrast to the classical dispersion relations where the gap fans in. The dispersion relations of Rossby waves shown in the lower‐left panel of Figure 2 are similar to those on the β‐plane of the classical theory. A comparison between the numerical solution and analytical approximations shows that the harmonic approximation has no relevance to these results. The results of this case are summarized in the lower‐left cell of Table 1.

##### *A narrow channel (large* α=500andU=400)

In a narrow channel the dispersion relations of the Poincaré waves, shown in the lower right panel of Figure 1, are somewhat distorted compared to their counterparts of the classical *f*‐plane theory with no mean flow. In contrast to that theory where all curves are convex, here the curves change their convexity from convex to concave as *k* increases, except for the first mode that remains concave at all *k*. The slope of an imaginary line that connects the ω‐values at the *k*‐values for which the convexity changes (i.e. inflection points) and crosses the Poincaré modes, is of order $\sqrt{\alpha \left(1-yU/\alpha \right)}$ with *y* between −1 and 0 and is similar to the additional “Kelvin‐like”/”anti‐Kelvin‐like” modes that appeared above for other αandU values. Near these inflection points, the gap between the curves of adjacent modes becomes smaller. The positive and negative modes are quite symmetric. Another piece of evidence for the existence of additional modes is that for each *k* there are two modes with eigenfunctions that have the same number of internal zero‐crossings (including no zero crossings). However, these additional positive and negative modes both have their maximum of η near y=1, in contrast to classical Kelvin/anti‐Kelvin waves in which each of these modes has it maximum near the opposite wall. The dispersion relations of Rossby waves are shown in the lower‐right panel of Figure 2, where it is also clear that the harmonic approximation is not relevant. The results of this case are summarized in the lower‐right cell of Table 1.

In all cases presented in this section of the *f*‐plane, reversing the direction of the mean flow (i.e. for negative *U*) yields the exact phase speeds shown above but with the opposite signs and the eigenfunctions are reflected with respect to the ordinate (results not shown). Thus, for westward‐flowing uniform mean flow, Rossby waves propagate eastward!

## 4 THE EFFECT OF A MEAN FLOW ON THE *β*‐PLANE

On the *β*‐plane, substituting the expression $\bar{h}=1+U/\alpha \left(\frac{\beta }{6}-y-\frac{1}{2}\beta y^{2}\right)$ (Equation (3)) into (8) yields:

(17) $$\begin{array}{cc} & \frac{d}{\mathrm{dy}}\left[\begin{array}{c} \begin{array}{c} V \end{array}\\ \begin{array}{c} \eta \end{array} \end{array}\right]=\\ & \frac{1}{c}\left[\begin{array}{cc} \left(1+\beta y\right)\left(1+\frac{c}{\left(1+U/\alpha \left(\frac{\beta }{6}-y-\frac{1}{2}\beta y^{2}\right)\right)}U/\alpha \right) & \alpha -\frac{c^{2}}{1+U/\alpha \left(\frac{\beta }{6}-y-\frac{1}{2}\beta y^{2}\right)}\\ \frac{\left(k^{2}c^{2}-{\left(1+\beta y\right)}^{2}\right)}{\alpha } & -\left(1+\beta y\right) \end{array}\right]\left[\begin{array}{c} \begin{array}{c} V \end{array}\\ \begin{array}{c} \eta \end{array} \end{array}\right]\;. \end{array}$$

Due to the complexity of this set, only numerical solutions are presented in this section.

We set (arbitrarily) the value of $\beta =\cot\phi_{0}\frac{L}{a}$ to 0.25, that is, a channel width, 2*L*, of $\frac{1}{2}a\approx$ 3,200 km at $\phi_{0}=45{}^{\circ}$, and calculate the solutions for the four pairs of α and U used in the previous (*f*‐plane) section.

Heuristic considerations suggest that for small β<1 and small U/α<1, their contributions will be additive since on the *f*‐plane the mean‐flow term U/α plays the role of *β*. Thus, in the presence of both mean‐flow and *β*, we might expect the zeroth order of the phase speed of Rossby waves to be similar to that on the *f*‐plane, Equation (13), by adding β to U/α in the nominator. This requires further assumptions such as assuming that $\bar{h}$= 1 but $\frac{d\bar{h}}{\mathrm{dy}}=-U/\alpha \neq 0$ and neglecting quadratic terms as $\beta^{2}$, βU/α, ${\left(U/\alpha \right)}^{2}$. Vallis (2017) derived such an expression for the phase speed using Quasi‐Geostrophic Shallow‐Water (QG‐SW) equations (see his equation 6.66a), which in terms of the scaling of the present study is:

(18) $$c_{n}^{\text{Rossby},\;\mathrm{QG}-\mathrm{SW}}=\frac{-\left(\beta +U/\alpha \right)}{k^{2}+\pi^{2}{\left(n+1\right)}^{2}/4+1/\alpha },$$

so it might be a basis for comparison. (Note that when β=0 this expression is exactly the approximate expression presented above, Equation (13), for the phase speed of Rossby waves on the *f*‐plane.) For the same reasons, we expect β to affect the phase speed of Poincaré waves only slightly.

**FIGURE 4:**
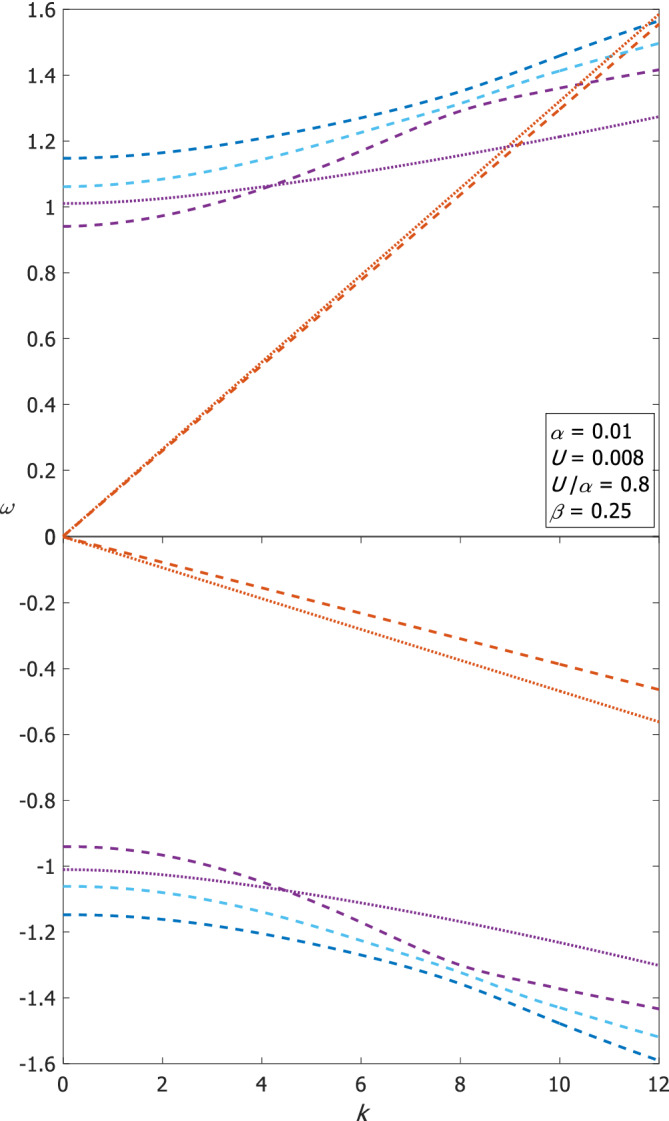
The dispersion relations of the first (i.e. *n* = 0, 1, 2) positive and negative Poincaré wave modes on the *β*‐plane (dashed lines, |ω_*n*_| increases with *n*) and the two additional “Kelvin‐like/anti‐Kelvin‐like” modes on the *β*‐plane (straight, dashed orange lines). Also shown, the counterparts on the *f*‐plane of the *n* = 0 Poincaré modes (dotted, purple lines) and the “Kelvin‐like”/“anti‐Kelvin‐like” modes (straight, dotted orange lines). The parameter values used in generating these curves are indicated on the figure [Colour figure can be viewed at wileyonlinelibrary.com]

**FIGURE 5:**
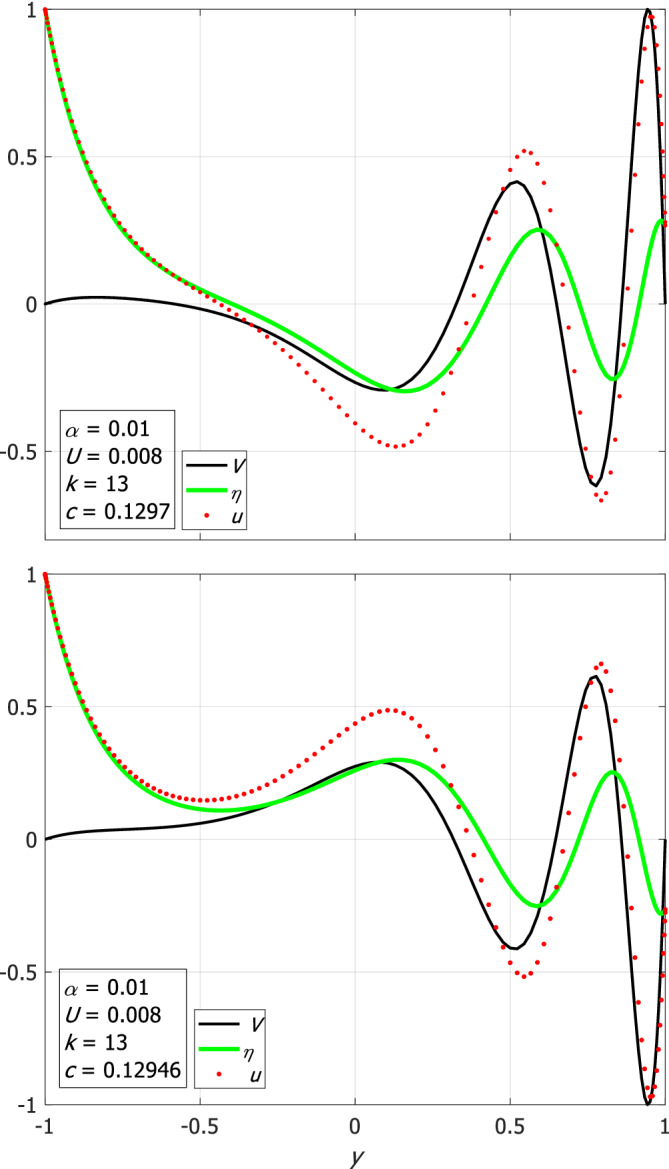
Examples of eigenfunctions of two modes with *k* = 13 and with nearly identical phase speeds of 0.13 and similar *V*‐eigenfunction. One mode is a “Kelvin‐like” wave and the other is a Poincaré mode. There is no way of confidently classifying which of them is a “Kelvin‐like” and which is a Poincaré mode. All eigenfunctions are normalized such that their maximum/minimum amplitude is 1/−1 [Colour figure can be viewed at wileyonlinelibrary.com]

For the most part, the structure of the dispersion relations of Poincaré waves is similar to the *f*‐plane while the frequency values change by less than 10% at most for *k* = 0 and *n* = 0. Figure 4 shows that in a wide channel (α=0.01) and for U=0.008 (*U*/α =0.8) the convexity of the dispersion curves of the first (i.e. *n* = 0, 1, 2) Poincaré wave modes (positive and negative) changes at k≈7 and the curves become bunched up around this *k* (dashed lines for the *β*‐plane and dotted lines for the *f*‐plane). The dispersion curves of the “Kelvin‐like” and “anti‐Kelvin‐like” modes are also similar to those on the *f*‐plane, but the “Kelvin‐like” wave crosses the Poincaré modes at higher *k*‐value on the *β*‐plane compared to the *f*‐plane. The corresponding phase speeds of the “Kelvin‐like” waves obey $c^{2}\approx \alpha +U\left(\frac{\beta }{6}-y-\frac{1}{2}\beta y^{2}\right)$ evaluated at y=−1 for the “Kelvin‐like” wave or at y=1 for the “anti‐Kelvin‐like” wave since the η‐eigenfunctions of these modes decay exponentially within a short distance from either of the channel walls for small *k* values. In the classical Kelvin wave theory the *y*‐dependent amplitudes of the η‐eigenfunctions do not depend on *k* and have no zero‐crossings. In contrast, here the amplitudes of the η‐eigenfunctions vary with *k* for large *k* (beyond the first intersection point with the inertia–gravity modes as can be noticed in Figure 4) and oscillate across the channel following the decay right near the wall. Though for large *k* the η‐eigenfunctions do not decay monotonically with distance from the wall, the above expression of *c* ^2^ with the substitution of y=1 or − 1 still provides an acceptable approximation to the numerically computed dispersion curves. Figure 5 shows an example for the eigenfunctions of two modes that have nearly identical phase speeds (corresponding to that of the “Kelvin‐like” wave, $c^{2}\approx \alpha +U\left(\frac{\beta }{6}-y-\frac{1}{2}\beta y^{2}\right)$, evaluated at y=−1) and similar *V*‐eigenfunction, while the η‐eigenfunctions are slightly different. The η‐eigenfunction of both the “Kelvin‐like” wave and one of the Poincaré modes (that cannot be distinguished from one another) decays from the y=−1 wall and then oscillates with further increase in *y*, which is in contrast to the behaviour of traditional Kelvin waves.

**FIGURE 6:**
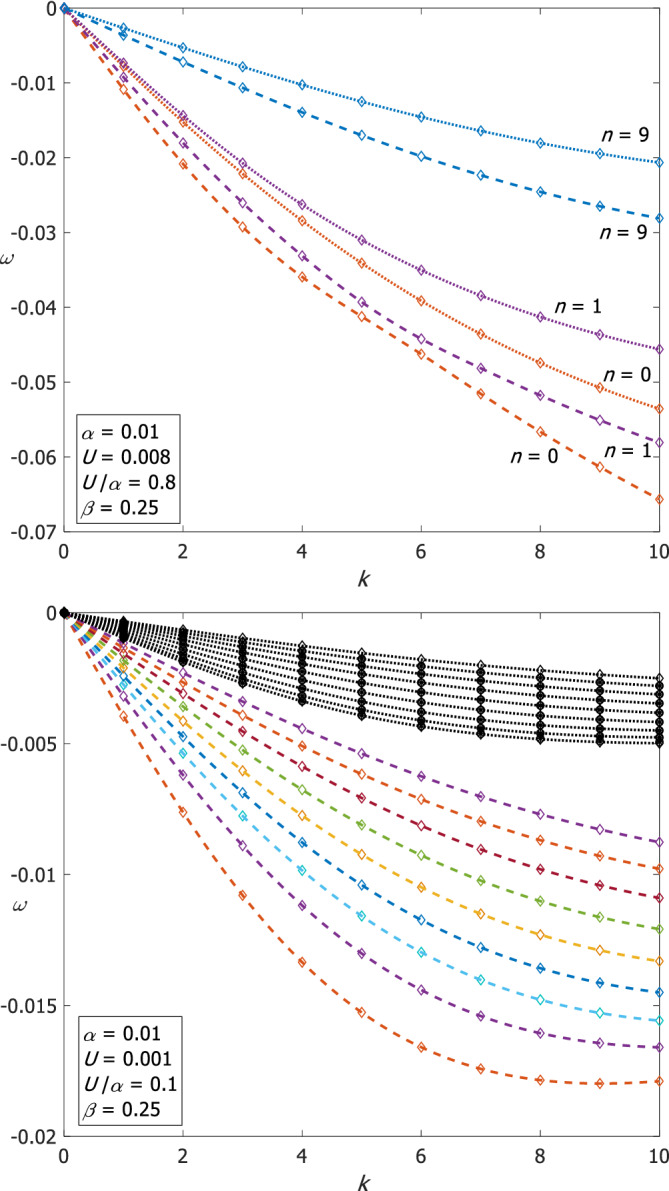
A comparison between the dispersion relations of Rossby waves on the *β*‐plane and on the *f*‐plane for the indicated parameter values, for large U/α (upper panel) and for small U/α (lower panel). Dashed lines denote the results on the β‐plane and dotted lines on the *f*‐plane [Colour figure can be viewed at wileyonlinelibrary.com]

The phase speeds of Rossby waves are larger (in absolute value) on the *β*‐plane compared with their counterparts on the *f*‐plane, as anticipated above. The differences between the frequency values on the *β*‐plane and those on the *f*‐plane are significant even when U/α is small. Figure 6 compares the dispersion relations of Rossby waves on the two planes for two cases. The upper panel shows the comparison for α=0.01 and U=0.008 (U/α=0.8) for *n* = 0, 1 and 9 where the difference is about 10% while the lower panel shows the comparison for α=0.01 and U=0.001 (*U*/α =0.1) for *n* = 0–8 where the difference exceeds 350% since β>U/α. Similar to Poincaré waves, the *n* = 0 Rossby mode changes its convexity at k≈5 for α=0.01 and for U=0.008 on the *β*‐plane.

**FIGURE 7:**
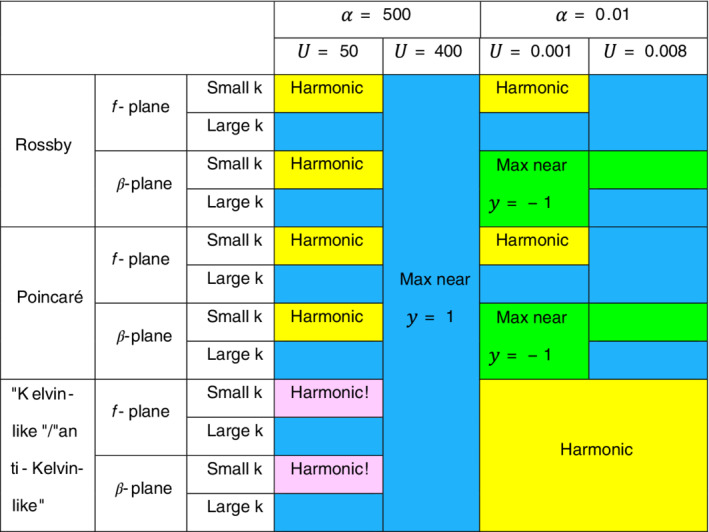
A table comparing the eigenfunctions' structure between the classical harmonic theory and the present study's set‐up with mean flow on either the *f*‐plane or the β‐plane. Yellow entries represent no difference between the classical harmonic theory and the present set‐up where the eigenfunctions of Rossby and Poincaré waves are sine/cosine and those of the Kelvin/anti‐Kelvin waves are exponential decay from either of the channel walls (recall: with no mean flow there are no Rossby waves on the *f*‐plane). Pink entries stand for “Kelvin‐like”/“anti‐Kelvin‐like” waves that are harmonic instead of exponential. Blue entries stand for cases in which the eigenfunctions are not harmonic and the maximum amplitude occurs near y=1. Green entries stand for cases in which the eigenfunction on the β‐plane with mean flow is neither harmonic nor similar to that on the *f*‐plane with mean flow, so the maximum amplitude occurs near y=−1 [Colour figure can be viewed at wileyonlinelibrary.com]

The eigenfunctions on the *β*‐plane differ from those on the *f*‐plane in a few cases only. The table shown in Figure 7 compares the characteristics of the eigenfunctions of the classical harmonic theory with those of the present study on both the *f*‐plane and the *β*‐plane for the four combinations of parameters. The eigenfunctions are harmonic (with maximum amplitude at y≈0 for the *n* = 0 mode) only for small *k* and when U/α is small. In all other cases the maximum amplitude gets closer to the y=1 channel wall and the eigenfunctions become more similar to the Airy function as *k* increases. In a wide channel (i.e. small α=0.01) the maximum amplitude is near y=−1 while for U=0.008 this maximum shifts from y≈−1 to y≈1 as *k* increases. This shift of the maximum amplitude is reflected also in the change of convexity of the dispersion relations discussed above. The reason for this shift might be associated with the effects that β and U have on the potential vorticity.

**FIGURE 8:**
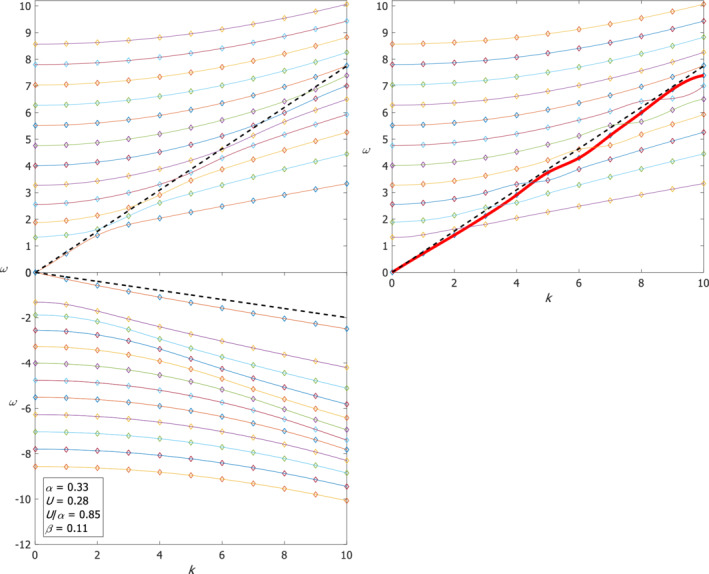
The dispersion relations of the Poincaré waves and two additional “Kelvin‐like” and “anti‐Kelvin‐like” for realistic values of the parameters indicated in the box. Dashed lines are analytic approximation of the “Kelvin/anti‐Kelvin‐like” dispersion relations. The right panel shows another way to connect the points to curves [Colour figure can be viewed at wileyonlinelibrary.com]

**FIGURE 9:**
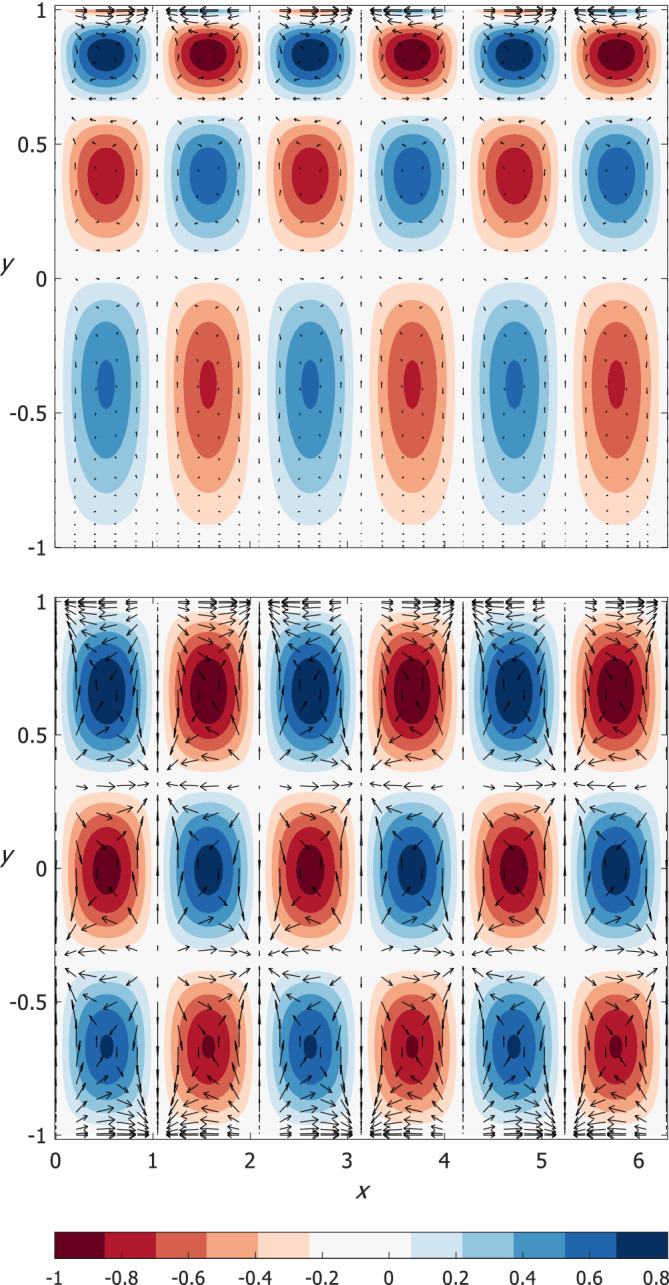
The eigenfunctions of the *k* = 3, *n* = 2 Rossby wave for realistic values of the parameters: α=0.33, β=0.11. Contours represent η level and arrows are the *u*,*v* velocity vectors. Upper panel: Realistic U=0.28. Lower panel: Harmonic eigenfunctions for U=0 [Colour figure can be viewed at wileyonlinelibrary.com]

The values of parameters for which the numerical solutions were calculated are somehow arbitrary since a wide or narrow channel does not really exist in the “real world”; therefore, we also examine the results for realistic values. For typical radius of deformation of about 400 km and half channel width of 700 km, α≈0.33 so the dimensional mean flow should be smaller than 23 m·s^−1^ according to condition (4). Such a restriction does not exist in QG and it emerges directly from the geostrophy, Equation (1). We choose the mean flow to be 20 m·s^−1^, that is, U≈0.28 so U/α=0.85 and the value of β (at $\phi_{0}=45{}^{\circ}$) is $\beta =\cot\phi_{0}\frac{L}{a}\approx 0.11$ which is quite small so the results are similar to those on the *f*‐plane. Figure 8 shows the dispersion relations of positive and negative Poincaré waves and the analytic approximation for the “Kelvin‐like” and “anti‐Kelvin‐like” waves (dashed lines), $c^{2}\approx \alpha +U\left(\frac{\beta }{6}-y-\frac{1}{2}\beta y^{2}\right)$ evaluated at y=−1 for the “Kelvin‐like” wave and y=1 for the “anti‐Kelvin‐like” wave. Since curves that belong to different wave types cross one another it is not obvious how the points that form the dispersion curves should be connected, and in the right panel of Figure 8 we show that one can draw an alternate curve for the “Kelvin‐like” wave by following the analytic estimate, (which yields the red line) thus distorting the other lines. The dispersion relations of Rossby waves do not match the harmonic approximation since U/α is large (results not shown). The variation of the eigenfunctions in both the zonal and meridional directions is shown in the upper panel of Figure 9 for *k* = 3 and *n* = 2 Rossby wave where the contours are the values of η and the arrows are the velocity vectors. These eigenfunctions are compared with their classical harmonic waves' counterparts (i.e. when *U* = 0) shown in the lower panel of Figure 9 and it is clear that there are significant differences in the meridional direction.

When the direction of the mean flow is reversed (i.e. for negative *U*) the phase speeds of the Poincaré and “Kelvin‐like” waves are very similar to those with positive *U* (where “Kelvin‐like” waves obey $c^{2}\approx \alpha +U\left(\frac{\beta }{6}-y-\frac{1}{2}\beta y^{2}\right)$ evaluated at y=−1 for the “Kelvin‐like” wave and y=1 for the “anti‐Kelvin‐like” wave). However, the phase speeds of Rossby waves change dramatically since the value of β+U/α changes appreciably. This is in contrast to the scenario on the *f*‐plane where negative *U* only changes the sign of the phase speeds. The eigenfunctions of the Poincaré and Rossby waves are reflected with respect to the ordinate such that the maximum amplitude occurs near y=−1 instead of y=1 (results not shown). A special case occurs when β=−U/α in which the Doppler‐shifted phase speed of the resulting Rossby waves is very close to, but does not equal, zero. In this case, there are two “families” of Rossby waves: for each *n* there is one member that propagates eastward and another that propagates westward at the same phase speed.

## 5 DISCUSSION

In this work we consider, for the first time, the effect of *uniform* mean flow on zonally propagating waves based on the RSWE (instead of the vorticity and QG equations) in which the height field is in geostrophic balance with the mean flow on the *f*‐plane and the *β*‐plane. This surface height gradient associated with the uniform mean flow plays the role of *β* and we show that it generates Rossby waves even on the *f*‐plane, in addition to Poincaré and “Kelvin‐like” waves whose phase speeds are not merely Doppler‐shifted from their *U* = 0 counterparts. The geostrophic relation between the mean flow and the *y*‐variation of the mean height implies a vorticity gradient due to the sloping surface that is analogous to that of bottom topography, β‐effect or mean shear which explains why the resulting phase speeds are modified by the mean flow by more than just a Doppler shift.

In contrast to the classical wave theory on the *β*‐plane, here, no Sturm–Liouville (or Schrödinger) equation applies so it is not clear whether the properties of the solutions of these equations on the β‐plane are relevant to the solutions of the problem studied here. However, we find that some of the properties are evident, like the discrete infinite number of Rossby and Poincaré modes that do not cross each other (but the gap between adjacent Poincaré modes does not decrease monotonically with *k*). The additional “Kelvin‐like” modes reported here do not have the classical properties of Kelvin waves of the traditional theory. The meridional structure of the solutions is not V≡0 (similar to V≠0 on the sphere, see Garfinkel *et al*., 2017) and η decays near one of the channel walls only for some of the combinations of α and U, so the identification of these waves by their *y*‐dependent eigenfunction (which depends on *k*) is not trivial. However, their phase speeds obey a modified version of the classical expression $c^{2}=\alpha$. For some α and U values and above a certain value of *k*, the “Kelvin‐like” dispersion curve crosses those of Poincaré modes and therefore there are two modes with the same number of internal zero‐crossings. In the case of a wide channel and large Rossby number (α=500 and U=400) the points of the dispersion relations of the Poincaré and “Kelvin‐like” modes are associated with curves according to their expected values but the “Kelvin‐like” modes cannot be clearly separated from the other modes. Rather it is the virtual linear curve that obeys the modified expression for the “Kelvin‐like” waves phase speed, so the curves above it are convex and those below it are concave (see lower‐right panel of Figure 1).

No instabilities were found in our numerical solutions even though the dispersion curve of the Kelvin wave intersects inertia–gravity (and Rossby) modes. This result is consistent with the results reported in Cohen *et al*. (2016) who studied the instabilities that develop in an upper‐layer vortex of a two‐layer ocean and concluded that instabilities develop only when wave‐modes in one layer intersect other wave‐modes in the other layer. Similar instability scenarios in a two‐layer ocean were also reported in Orlanski (1968), Sakai (1989) and Iga (1993).

**FIGURE 10:**
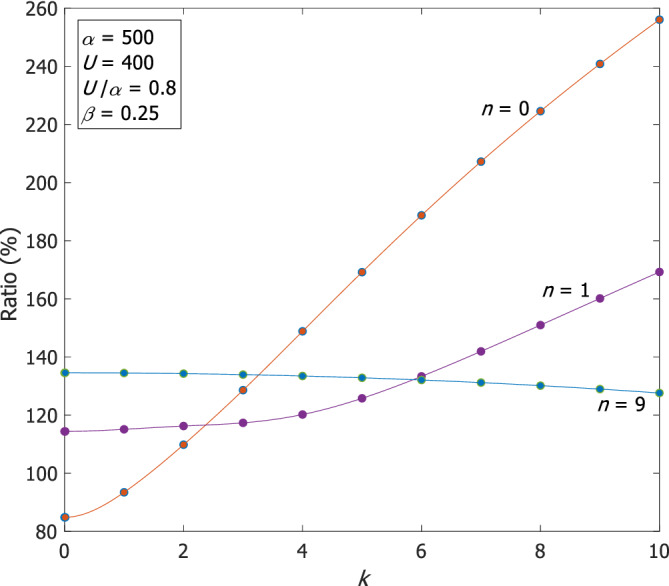
The ratio between our numerical phase speeds of Rossby waves on the β‐plane and the QG‐SW phase speeds for *n* = 0, 1, 9 for α=500 and U=400 [Colour figure can be viewed at wileyonlinelibrary.com]

A comparison of our numerical results of the phase speeds of Rossby waves on the *β*‐plane with the corresponding analytic solution of the QG‐SW theory, Equation (18), shows that for small *U*/α the QG‐SW phase speeds differ from our numerical results by less than 15%. On the other hand, for large *U*/α and large α (i.e. narrow channel) the differences are very significant. Figure 10 shows the ratio between our phase speeds and those of QG‐SW for *n* = 0, 1 and 9, α=500 and U=400, where for *n* = 0, *k* = 10 our phase speed is faster by a factor of more than 2.5 compared to that of the QG‐SW solution.

**FIGURE 11:**
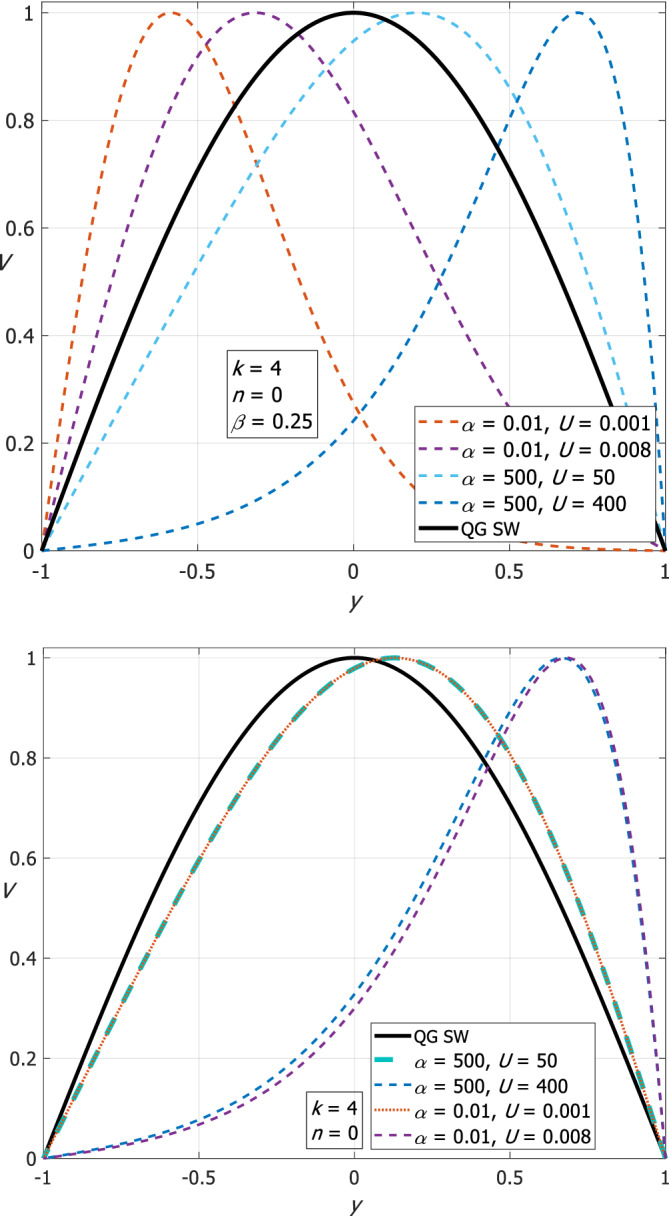
The *V*‐eigenfunction (normalized such that the maximum is 1) of Rossby waves for *k* = 4 and *n* = 0 in the case where the mean flow is consistently accounted for in the present study for the four combinations of α and U on the β‐plane (upper panel) and on the *f*‐plane (lower panel). *V* of the QG‐SW theory is a simple cosine (solid black) and independent of α and U [Colour figure can be viewed at wileyonlinelibrary.com]

Even though the QG‐SW theory describes the phase speed of Rossby waves quite well at small U/α, it fails to account for the meridional structure of the solutions. Figure 11 shows the *n* = 0 meridional structure functions of Rossby waves for four combinations of the values of α and U (dashed lines) on the β‐plane (upper panel) and on the *f*‐plane (lower panel). The meridional structure function of the QG‐SW theory (solid black) is a simple cosine that does not depend on α and U. A comparison of the dashed lines with the solid line clarifies that the structure function of the QG‐SW theory differs appreciably from the actual numerical solution, where the peak is shifted off mid‐channel (towards −1 or 1). In contrast, the corresponding QG‐SW phase speeds are very close to our numerical phase speeds in some of the combinations (on the β‐plane about 1% for α=500 and U=50 and for α=0.01 and U=0.008, about 12% for α=0.01 and U=0.001, and about 48% for α=500 and U=400, all for *k* = 4 and *n* = 0).

In the trapped wave theory, the *y*‐dependence of $f\left(y\right)=f_{0}+\beta y$ is consistently accounted for throughout so the equations have *y*‐dependent coefficients that leads to the solution of a trapped Airy function. In the set‐up of the present study where the mean flow is uniform, there are additional terms associated with the $\bar{h}\left(y\right)$ expression so the meridional structure of Poincaré and Rossby waves is also not harmonic (except for small *k* and small *U*/α) i.e. trapped (so they are approximated by Airy functions in some limit) even on the *f*‐plane. In contrast, in the QG‐SW theory the equations only contain the two constants $f_{0}$ and β (so the equations are in fact constant‐coefficient equations) which is why this theory cannot be tuned to fit trapped waves.

In the present study we show that increasing α by more than 4 orders of magnitudes (to unrealistic values) does not change the results drastically as long as the ratio *U*/α is conserved, and so the dominant parameter is the ratio between U and α rather than their individual values. It is also evident from the lower (*f*‐plane) panel of Figure 11 that for the same U/α but different values of α and U, the curves are very close to each other and the structure of the *V* eigenfunction changes from harmonic to trapped as *U*/α increases.

In the atmosphere, strong currents like the jet stream have a strong shear that generates Rossby waves by the gradient of relative vorticity. Since the flow in the jet stream is not uniform our results do not apply directly to this scenario and the extension of our results to non‐uniform mean zonal flow is left for a future work.

**FIGURE 12:**
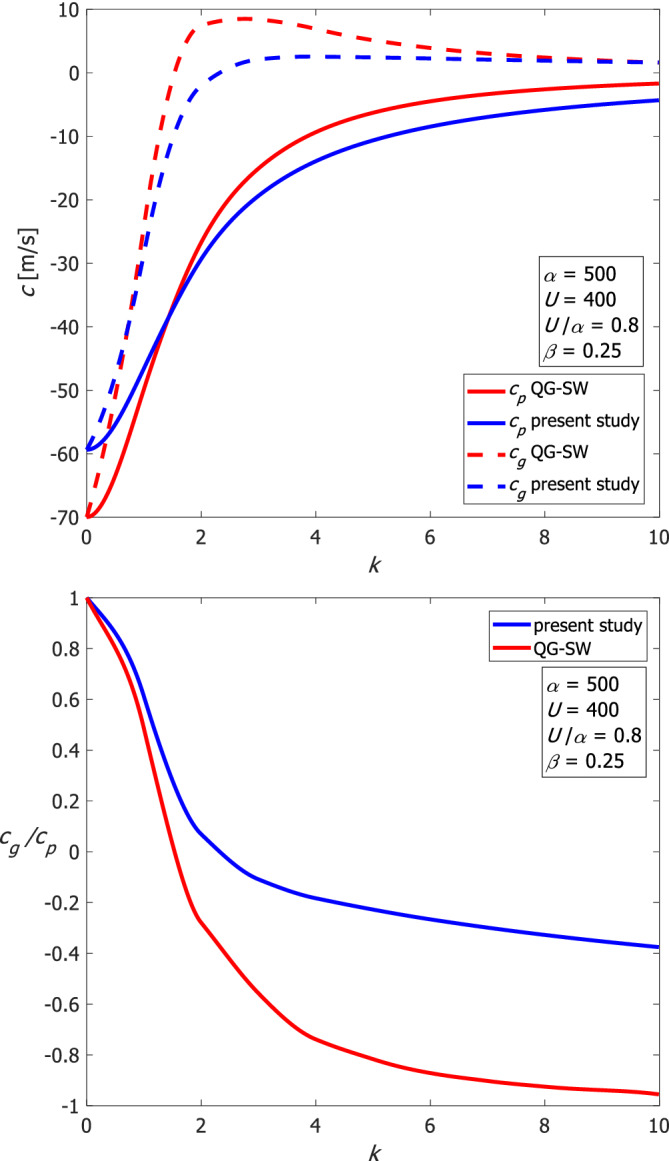
Upper panel: The dimensional zonal group velocity (dashed) and the phase speed (solid) of Rossby waves for both QG‐SW and our solutions for *n* = 0 for α=500 and U=400. Lower panel: The ratio of the zonal group velocity to the phase speed of Rossby waves for both QG‐SW and the solutions of the present study for the same parameters [Colour figure can be viewed at wileyonlinelibrary.com]

Our results also have implications for downstream development of low‐pressure systems (Chang, 1993), in which the envelope of Rossby wave activity is observed to propagate downstream faster than individual wave‐modes. This effect has been interpreted as due to the zonal group velocity exceeding the phase speed (Cressman, 1948; Blackmon *et al*., 1984; Hakim, 2003; Danielson *et al*., 2006); however, these previous studies employed the QG theory to describe wave propagation. The present study has demonstrated that the zonal phase speed (and hence also the group velocity) differs from that in QG theory. Specifically, phase speeds in the theory developed here can exceed those of the QG theory by a factor of 2. This difference is most pronounced for large values of *U*, and also for a narrow channel. The dimensional zonal group velocity and phase speed are shown in the upper panel of Figure 12 for QG‐SW theory and for our solutions. The ratio of the zonal group velocity to the phase speed is shown in the lower panel of Figure 12. The zonal group velocity is typically eastward even when the phase speed is westward for both theories for synoptic waves, and hence downstream development should be expected to occur. However, the magnitude of this effect differs: in QG theory the magnitude is similar and just the sign differs (so downstream development should be very fast); in the more accurate theory of the present study, downstream development is much more gradual. These results also have implications for the group velocity of Rossby wave packets (Wirth *et al*., 2018), as the group velocity in our revised theory is slower than that of the QG theory.

## CONFLICT OF INTEREST

The authors declare that they have no conflict of interest.

## References

[qj4107-bib-0001] Blackmon, M.L. , Lee, Y.H. , Wallace, J.M. and Hsu, H.‐H. (1984) Time variation of 500 mb height fluctuations with long, intermediate and short time scales as deduced from lag‐correlation statistics. Journal of the Atmospheric Sciences, 41(6), 981–991. 10.1175/1520-0469(1984)041<0981:TVOMHF>2.0.CO;2.

[qj4107-bib-0002] Chang, E.K.M. (1993) Downstream development of baroclinic waves as inferred from regression analysis. Journal of the Atmospheric Sciences, 50(13), 2038–2053.

[qj4107-bib-0003] Cohen, Y. , Dvorkin, Y. and Paldor, N. (2016) On the stability of outcropping eddies in a constant‐PV ocean. Quarterly Journal of the Royal Meteorological Society, 142(698), 1920–1928. 10.1002/qj.2785.

[qj4107-bib-0004] Cressman, G.P. (1948) On the forecasting of long waves in the upper westerlies. Journal of Meteorology, 5(2), 44–57.

[qj4107-bib-0005] Danielson, R.E. , Gyakum, J.R. and Straub, D.N. (2006) A case study of downstream baroclinic development over the North Pacific Ocean. Part II: Diagnoses of eddy energy and wave activity. Monthly Weather Review, 134(5), 1549–1567.

[qj4107-bib-0006] De‐Leon, Y. and Paldor, N. (2009) Linear waves in midlatitudes on the rotating spherical Earth. Journal of Physical Oceanography, 39, 3204–3215. 10.1175/2009JPO4083.1.

[qj4107-bib-0007] Garfinkel, C.I. , Fouxon, I. , Shamir, O. and Paldor, N. (2017) Classification of eastward propagating waves on the spherical Earth. Quarterly Journal of the Royal Meteorological Society, 143(704), 1554–1564. 10.1002/qj.3025. 31423027 PMC6686444

[qj4107-bib-0008] Gildor, H. , Paldor, N. and Ben‐Shushan, S. (2016) Numerical simulation of harmonic, and trapped, Rossby waves in a channel on the midlatitude *β*‐plane. Quarterly Journal of the Royal Meteorological Society, 142(699), 2292–2299. 10.1002/qj.2820.

[qj4107-bib-0009] Hakim, G.J. (2003) Developing wave packets in the North Pacific storm track. Monthly Weather Review, 131(11), 2824–2837.

[qj4107-bib-0010] Iga, K. (1993) Reconsideration of Orlanski's instability theory of frontal waves. Journal of Fluid Mechanics, 255, 213–236. 10.1017/S0022112093002460.

[qj4107-bib-0011] Orlanski, I. (1968) Instability of frontal waves. Journal of the Atmospheric Sciences, 25, 178–200.

[qj4107-bib-0012] Paldor, N. and Dvorkin, Y. (2006) Barotropic instability of a zonal jet: from nondivergent perturbations on the *β* plane to divergent perturbations on a sphere. Journal of Physical Oceanography, 36(12), 2271–2282.

[qj4107-bib-0013] Paldor, N. and Sigalov, A. (2008) Trapped waves on the mid‐latitude *β*‐plane. Tellus A, 60, 742–748. 10.1111/j.1600-0870.2008.00332.x.

[qj4107-bib-0014] Paldor, N. , Rubin, S. and Mariano, A.J. (2007) A consistent theory for linear waves of the shallow‐water equations on a rotating plane in midlatitudes. Journal of Physical Oceanography, 37, 115–128. 10.1175/JPO2986.1.

[qj4107-bib-0015] Pedlosky, J. (1987) Geophysical Fluid Dynamics. New York, NY: Springer‐Verlag.

[qj4107-bib-0016] Sakai, S. (1989) Rossby–Kelvin instability: a new type of ageostrophic instability caused by a resonance between Rossby waves and gravity waves. Journal of Fluid Mechanics, 202, 149–176. 10.1017/S0022112089001138.

[qj4107-bib-0017] Vallis, G.K. (2017) Atmospheric and Oceanic Fluid Dynamics: Fundamentals and large‐scale circulation, 2nd edition. Cambridge, UK: Cambridge University Press. 10.1017/9781107588417.

[qj4107-bib-0018] Wirth, V. , Riemer, M. , Chang, E.K.M. and Martius, O. (2018) Rossby wave packets on the midlatitude waveguide – a review. Monthly Weather Review, 146(7), 1965–2001.

